# Misidentification and misreporting of antibiotic resistance in *Kluyvera* bacteremia by blood culture molecular identification panels

**DOI:** 10.1128/spectrum.00542-24

**Published:** 2024-04-23

**Authors:** Darrick Vatterrodt, Janelle Lee, Dooil Ho, Craig Stevig, Siu-Kei Chow

**Affiliations:** 1 Department of Microbiology, MultiCare Health System, Tacoma, Washington, USA; 2 Department of Microbiology, Veterans Administration Puget Sound Health Care System, Seattle, Washington, USA; Montefiore Medical Center and Albert Einstein College of Medicine, Bronx, New York, USA

**Keywords:** MALDI-TOF MS, PCR, sepsis, multiplex, syndromic, rapid diagnostics, antimicrobial resistance

## Abstract

The use of molecular identification panels has advanced the diagnosis for blood stream infections with fast turnaround time and high accuracy. Yet, this technology cannot completely replace conventional blood culture and standardized antibiotic susceptibility testing (AST) given its limitations and occasional false results. Here we present two cases of bacteremia caused by *Kluyvera*. Its identification and antibiotic resistance were at least partially mispresented by blood culture molecular identification panels on ePlex, Verigene, and Biofire. The detection of CTX-M resistance marker did not align with the susceptibility to the third generation cephalosporins among a wide range of antibiotics for this organism. Conventional extended-spectrum beta-lactamase (ESBL) testing was used to confirm the absence of ESBL. Caution should be taken when managing cases with CTX-M or ESBL detection in blood culture caused by uncommon pathogens. Conventional culture with microbial identification and standardized AST should continue to be the gold standard for routine patient care.

**IMPORTANCE:**

This is the first report that highlights the limitations of blood culture molecular identification panels on identifying *Kluyvera* and its associated antibiotic resistance patterns. Both the false identification and overreporting of antibiotic resistance could mislead the treatment for bacteremia caused by this pathogen. Patient isolation could have been avoided due to the lack of extended-spectrum beta-lactamase (ESBL) activity of the organism. This report emphasizes the importance of confirming rapid identification and antibiotic resistance by molecular technologies with standardized methods. It also provides insight into the development of new diagnostic panels.

## OBSERVATION

Molecular identification panels for positive blood culture have been widely used to help with fast and accurate diagnosis for blood stream infections (BSIs). When being used appropriately with prompt responses by providers and infection preventionists, these panels improve antibiotic stewardship, facilitate patient isolation or deisolation, and reduce length of stay (1–3). Yet, cross-reactivity between species may lead to misidentification (4). An atypical false negative result of a common pathogen using blood culture panel has been reported (5).

### Case reports

#### Case 1

An 81-year-old female with a history of irritable bowel syndrome, chronic kidney disease, and recurrent urinary tract infection presented to the emergency department (ED) of Deaconess Hospital (Spokane, WA) with severe sepsis and hypotension. She reported nausea and emesis, but denied diarrhea and fever. Her white blood cell count and serum lactate level were high (13,400 cells/µL and 3.0 mmol/L, respectively). She presented with transaminitis (2,062 IU/L for aspartate aminotransferase, 1,324 IU/L for alanine transaminase (ALT), 633 IU/L for alkaline phosphatase (ALP)) that was associated with shock liver. Her urine culture grew mixed normal flora, while her two sets of blood culture grew Gram-negative bacilli within 1 day of incubation. The ePlex BCID-GN PCR test (Roche Diagnostics, Indianapolis, IN) was performed on the positive blood culture with the detection of *Enterobacter cloacae* complex and CTX-M (Table 1). The patient was first treated empirically with ceftriaxone (2 g, daily), and her regimen was switched to meropenem (1,000 mg, every 12 hours) upon the release of CTX-M PCR result. Standardized microbial identification and AST testing by the Negative Urine Combo 102 panel on MicroScan Walkaway plus (Beckman Coulter, Brea, CA) identified the organism as *Kluyvera ascorbata* that is susceptible to a wide range of antibiotics including third and fourth generation cephalosporins except for ceftriaxone (Table 2). Treatment with meropenem continued for 3 days, followed by ertapenem (1 g, daily) for 1 day. Upon discharge, the patient was prescribed ciprofloxacin (500 mg, every 12 hours) for 10 days.

**TABLE 1:** Microbial identification by molecular panels and standardized methods^*d*^

Test methodology	Case 1		Case 2	
	Identification	Resistance marker	Identification	Resistance marker
ePlex BCID-GN^*a*^	*Enterobacter cloacae* complex	CTX-M	*Enterobacter cloacae* complex	CTX-M
Verigene BC-GN^*a*^	*Klebsiella oxytoca*	CTX-M	*Klebsiella oxytoca*	CTX-M
Biofire BCID2^*a*^	Enterobacterales	CTX-M	Enterobacterales	CTX-M
MALDI-TOF MS (score)	*Kluyvera ascorbata* (2.29)^*b*^/ *Kluyvera cryocrescens* (1.80)^*c*^	NA	*Kluyvera ascorbata* (2.08) ^*b*^/ *Kluyvera cryocrescens* (1.82)^*c*^	NA
BD Phoenix NMIC/ID-307 (% confidence)	*Enterobacter cloacae* (90%)	NA	*Kluyvera intermedia* (99%)	NA
MicroScan Negative Urine Combo 102	*Kluyvera ascorbata*	NA	*Kluyvera ascorbata*	NA

^*a*^ Blood culture molecular panels were performed on positive blood culture bottles from original patient collection and by spiking pure Gram-negative bacterial isolate from subcultures, respectively.

^*b*^ MALDI Biotyper Compass Version 4.1.100.

^*c*^ MALDI Biotyper CA Version 3.2.

^*d*^ NA, not applicable.

**TABLE 2:** Antibiotic susceptibility testing by standardized methods^*e*^

Antibiotic	Case 1				Case 2			
	Phoenix	MicroScan	Sensititre	Interpretation^*b*^	Phoenix	MicroScan	Sensititre	Interpretation^*b*^
Ampicillin	>16	>16	>16	R	16	16	>16	I/R
Ampicillin/Sulbactam	4	≤4	≤4	S	8	≤4	≤4	S
Piperacillin/Tazobactam	4	≤8	≤8	S	8	≤8	≤8	S
Aztreonam	≤2	≤4	≤1	S	≤2	≤4	≤1	S
Cefazolin	>16	>16	>16	R	>16	16	>16	R
Ceftazidime	≤2	≤1	≤1	S	≤2	≤1	≤1	S
Ceftriaxone	≤1	>2^*c*^	≤0.5	S/R	≤1	≤1	≤0.5	S
Cefepime	≤1	≤2	≤2	S	≤1	≤2	≤2	S
Ertapenem	≤0.25	≤0.5	≤0.25	S	≤0.25	≤0.5	≤0.25	S
Meropenem	≤0.5	≤1	NT	S	≤0.5	≤1	NT	S
Gentamicin	≤2	≤2	≤2	S	≤2	≤2	≤2	S
Tobramycin	≤2	≤2	≤2	S	≤2	≤2	4	S/I
Amikacin	≤8	NT	≤8	S	≤8	NT	≤8	S
Ciprofloxacin	≤0.25	≤0.25	≤0.25	S	≤0.25	≤0.25	≤0.25	S
Levofloxacin	≤0.5	NT	≤0.5	S	≤0.5	NT	≤0.5	S
Trimethoprim/Sulfamethoxazole	≤0.5	≤2	≤2	S	≤0.5	≤2	≤2	S
Tetracycline	≤2	≤4	NT	S	>8	8	NT	R
Minocycline	2	NT	2	S	>8	NT	>8	R
Etest of ceftriaxone	1^*d*^				NT			
ESBL confirmation^*a*^	negative				negative			

^*a*^ ESBL confirmatory testing was based on the CLSI recommendation for non-*Kluyvera* Enterobacterales. Disk diffusion using ceftazidime and ceftazidime-clavulanate, cefotaxime and cefotaxime-clavulanate was performed. A <5-millimeter increase in a zone diameter for either antibiotic tested in combination with clavulanate versus the zone diameter for the antibiotic alone indicates the lack of ESBL.

^*b*^ Based on CLSI M100 Table 2A on zone diameter and MIC breakpoints for Enterobacterales (6).

^*c*^ Resistance to ceftriaxone on MicroScan was consistent and confirmed with three independent runs.

^*d*^ Etest was repeated twice with the same MIC result .

^*e*^ MIC, minimum inhibitory concentration; NT, not tested; S, susceptible; I, intermediate; R, resistant.

#### Case 2

An 84-year-old male with a history of chronic obstructive pulmonary disease (COPD), tonsillar cancer in remission, and on-going decubitus ulcer presented to the ED of Good Samaritan Hospital (Puyallup, WA) with rigors and weakness. He was febrile (102.6°F) and septic on admission, with a white blood cell count (23,200 cells/µL) and slightly elevated liver function tests (47 IU/L for aspartate aminotransferase, 223 IU/L for ALT, 74 IU/L for ALP). His chest computed tomography (CT) scan result revealed cholecystitis with concern for gangrene, followed by a surgical drain placement into a ruptured gallbladder. The patient was treated empirically with ceftriaxone (2 g, daily) for one day then with piperacillin/tazobactam (3.375 g, every 8 hours). Two sets of blood cultures were positive with Gram-negative bacilli, identified by BC-GN PCR panel on Verigene (DiaSorin, Cypress, CA) as *Klebsiella oxytoca* with CTX-M. One of four blood culture bottles also grew methicillin-resistant *Staphylococcus aureus* (MRSA). Consequently, his antibiotic was switched to vancomycin (1,750 mg, every 24 hours) and meropenem (500 mg, every 6 hours). Standardized microbial identification and AST testing were performed using matrix-assisted laser desorption ionization-time of flight mass spectrometry (MALDI-TOF MS) (Bruker, Billerica, MA) and NMIC/ID-307 on BD Phoenix (Becton, Dickinson and Company, Franklin Lakes, NJ), respectively. His culture from decubitus ulcer grew *Escherichia coli* and *Enterococcus faecalis*, which was determined to contribute insignificantly to his symptoms. His drain culture grew mixed Gram-negative bacilli but in the absence of *K. oxytoca*. The Gram-negative organism from the blood culture was identified by MALDI-TOF MS as *K. ascorbata* (Table 1). Standardized AST and ESBL confirmation test showed susceptibility to all tested third and fourth generation cephalosporins (Table 2). The patient improved and, upon discharge, continued the course on vancomycin and meropenem for 6 weeks. During his follow-up visit 6 weeks post-discharge, the patient appeared well with no major concerns.

#### Further studies

To better evaluate the performance of blood culture molecular identification panels and compare with standardized identification/AST methods, we tested the positive blood culture bottles and *Kluyvera* isolates from both cases on a series of platforms. Positive blood culture bottles, from the original patient collections as well as by spiking of pure Gram-negative isolate from subcultures, were tested on ePlex, Verigene, and Biofire (bioMérieux, Durham, NC) with BCID-GN, BC-GN, and BCID2 panels, respectively. The identification of the organism cultured on sheep blood agar was performed using MALDI-TOF MS and the Gram-negative identification panels on MicroScan (Negative Urine Combo 102), and Phoenix (NMIC/ID-307). Standardized AST was performed on MicroScan (Negative Urine Combo 102), Phoenix (NMIC-306, NMIC/ID-307), and on Sensititre (Gram Negative GN7F AST Plate) (Thermo Fisher Scientific, Waltham, MA). All tests were performed according to the manufacturers’ instructions. The identification results were shown in Table 1. Discrepancies were noticed across the molecular panels but not between the two samples. The ePlex and Verigene panels misidentified the organism as *E. cloacae* complex and *K. oxytoca*, respectively, while the Biofire panel identified it as a member of Enterobacterales but not to the genus/species level. All three molecular panels detected CTX-M as the resistance marker. The molecular test results of positive bottles between patient collection and spiking were identical. In Case 1, MALDI-TOF MS and MicroScan identified the organism as *K. ascorbata,* whereas Phoenix called it *E. cloacae* with 90% confidence. In Case 2, the three platforms had an agreeable identification of *Kluyvera* species. The standardized AST methods revealed that the two *Kluyvera* isolates were generally susceptible to a variety of antibiotics except for ampicillin and cefazolin following the interpretation guideline for Enterobacterales per Clinical and Laboratory Standards Institute (CLSI) (Table 2) (6). Interestingly, *K. ascorbata* in Case 1 showed resistance to ceftriaxone with minimum inhibitory concentration of greater than 2 µg/mL on MicroScan repeatedly, although Etest and the remaining two platforms confirmed its susceptibility. The ESBL confirmation test indicated the lack of ESBL in both isolates, contrasting the detection of CTX-M by all molecular panels.

## DISCUSSION

*Kluyvera* is an uncommon pathogen that causes bacteremia, wound infection, urinary tract infection, and cholangitis (7–12). This organism is most well-known and studied for its chromosomal *bla*_CTX-M_ as the progenitor of CTX-M-type β-lactamases found in other Enterobacterales species (13–15). *Kluyvera* is resistant to ampicillin, first and second generation cephalosporins, but mostly susceptible to third and fourth generation cephalosporins. Multidrug resistance to cephalosporins is uncommon but has been reported (9, 10, 16). Recent studies describe its acquired resistance to carbapenems (17, 18). Carbapenems and quinolones were used more often in cases where multidrug-resistant (MDR) *Kluyvera* strains were involved (9, 10, 16). In contrast, cephalosporins, aminoglycosides, and piperacillin/tazobactam were usually used to treat non-MDR strains (8–10). In our cases, both patients were treated with carbapenems after the identification of CTX-M by the molecular panels, which is a common and acceptable practice for antimicrobial-resistant Enterobacterales (19).

Our study highlighted the misidentification and detection of CTX-M in *Kluyvera* by blood culture molecular identification panels whereas demonstrating susceptibility to third and fourth generation cephalosporins without ESBL activity. *Kluyvera* reclassified as a new genus in 1981 is phylogenetically close to *Enterobacter*, *Klebsiella,* and *Citrobacter* (20), which explains the misidentification on the molecular panels. In our cases, MALDI-TOF MS and MicroScan were able to identify the isolates as *Kluyvera* correctly, but BD Phoenix missed one of the two identifications. Our standardized AST results contrasted with the CTX-M detection by the molecular panels, suggesting the lack of β-lactamase and its activity. In an environmental study, Tanner et al. identified and characterized 31 bacterial strains with ESBL- and carbapenemase-type genes, in which five *Kluyvera* strains (three *Kluyvera georgiana*, one *K*. *ascorbata*, and one unspecified *Kluyvera* species) were included (21). The gene sequence homology to the corresponding CTX-M genes of interest ranged from 84% to 100%. Even positive with *bla*_CTX-M_, four of the five strains were susceptible to third and fourth generation cephalosporins. A clinical strain of *K. georgiana* was shown to possess chromosome-encoded CTX-M-78 β-lactamase, and was susceptible to cefotaxime, ceftazidime, and cefepime (22). Taken together, standardized AST is a better method to examine antibiotic resistance of *Kluyvera* compared to Enterobacterales species with plasmid-coded CTX-M β-lactamases.

Misidentification of pathogens and miscalling of antibiotic resistance in the setting of bacteremia could cause significant harm to the patient. Clinicians and laboratory professionals should be aware of these limitations of molecular technologies for *Kluyvera* to provide accurate result interpretation and good patient care.
